# Foreign Body Granulomas Induced by Intramuscular Leuprorelin Acetate Injection for Prostate Cancer: Clinical Mimics of Soft Tissue Sarcoma

**DOI:** 10.1155/2015/947040

**Published:** 2015-03-31

**Authors:** Khin Thway, Dirk C. Strauss, Myles J. Smith, Cyril Fisher

**Affiliations:** Sarcoma Unit, Royal Marsden Hospital, London SW3 6JJ, UK

## Abstract

We describe two cases of florid, foreign body granulomatous reaction occurring in the upper arms of males in their eighth decade, who were undergoing treatment with depot injection of leuprorelin acetate for prostatic carcinoma. These patients presented with rapidly enlarging extremity soft tissue masses and were referred to a tertiary sarcoma center with clinical suspicion of a primary soft tissue neoplasm. The occurrence of injection site granulomas secondary to leuprorelin acetate administration is rarely known outside the urological and dermatological communities, and their recognition is important due to the spectrum of clinical differential diagnoses and potential for diagnostic confusion with metastatic prostatic cancer and primary sarcoma and in order to avoid unnecessary stress and clinical intervention for patients.

## 1. Introduction

Leuprorelin acetate is a synthetic analogue of gonadotropin-releasing hormone (GnRH)/luteinizing hormone-releasing hormone (LHRH), which is indicated in the treatment of prostatic cancer, and is injectable as a depot formulation for long term, controlled drug release. It can cause florid granulomatous inflammation at the site of subcutaneous or intramuscular injection, which can present clinically as a rapidly enlarging soft tissue mass. We describe two cases, occurring either subcutaneously or deeply within the skeletal muscle of the upper arms of males in their eighth decades, who were undergoing androgen deprivation therapy for prostatic carcinoma with depot injection of leuprorelin acetate and who were referred to a tertiary sarcoma center with the suspicion of a soft tissue neoplasm. The occurrence of injection site granulomas secondary to leuprorelin acetate is little known outside the urological and dermatological communities. As these lesions can occur deeply and their clinical and radiological features can be concerning for malignancy, we highlight the importance of recognizing this phenomenon and of clinical correlation in diagnosis.

## 2. Case Report

**Figure 1:**
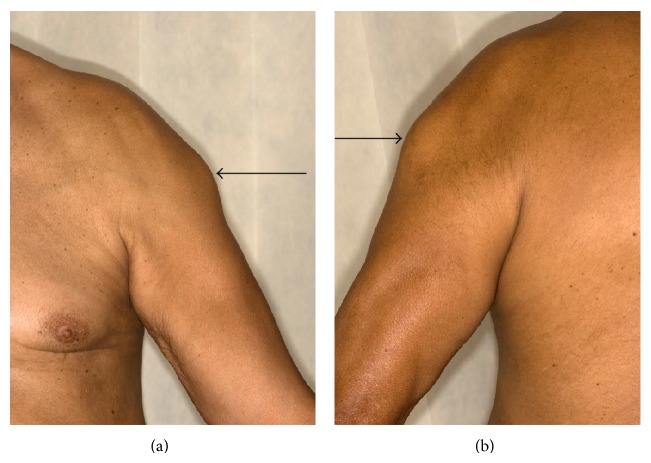
Clinically, the lesion was a relatively well defined, firm approximately 7 cm mass over the left biceps muscle, fixed to the underlying structures. No erythema of the overlying skin is seen in this example (case 2).

**Figure 2:**
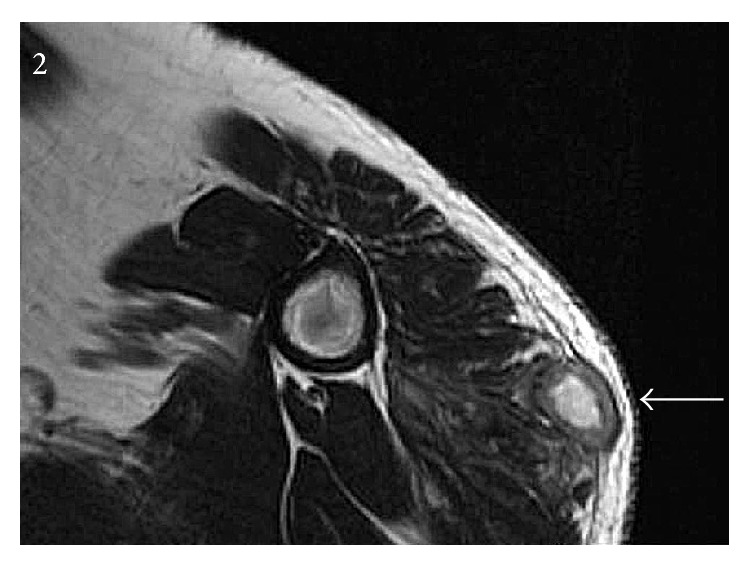
Magnetic resonance imaging (MRI) shows a complex, infiltrative maximally 9 cm mass in the left deltoid, extending from subcutaneous to deep tissues to abut the humerus, with underlying normal marrow. The lesion is composed of interlinked foci of abnormal tissue with intense central enhancement, suggesting necrosis.

The patients were 73- and 75-year-old males. Case 1 presented with an 11-week history of sudden onset, painful swelling of the right upper arm. He was referred to our sarcoma unit with the clinical and radiological suspicion of sarcoma. He had been diagnosed with T3bN0M0 Gleason score 4 + 5 prostatic adenocarcinoma 6 months prior to this, for which he had elected for systemic treatment. LHRH injections were commenced 3 months prior to presentation to sarcoma unit, and he was currently treated with leuprorelin acetate injection (brand name Prostap) at a dose of 11.25 mg once every 3 months. He had no other past medical history and no known history of travel or infection. On examination, there was an approximately 5 cm hard mass in the distal right biceps, just above the tendon. Clinically, the differential diagnoses were of organized hematoma, previous biceps muscle tear with healing by fibrosis, and myositis ossificans. Magnetic resonance imaging (MRI) showed a maximally 5.4 cm, ill-defined heterogeneous mass arising within the distal right biceps muscle, just proximal to the tendon, with peripheral enhancement and slightly increased T2/T1 signal relative to skeletal muscle, which was radiologically concerning for sarcoma. No evidence of disease was present elsewhere. Two needle core biopsies (initial and repeat) were performed. Six weeks after the initial biopsy and following the histological result for both biopsies, the patient was well and his right arm lesion was seen to be resolving. At this appointment, he was also noted to have a severe inflammatory reaction involving the skin and subcutis following a subsequent injection on the contralateral (left) upper arm. It was unknown whether the leuprorelin injections had been intramuscular or subcutaneous, but it was considered that he had intramuscular injection at the time of the first biopsy (with the reaction involving the biceps muscle) and then subcutaneous injections at the time of the second, which caused reaction in the superficial tissues. He was discharged from the sarcoma clinic; treatment with leuprorelin was stopped and he underwent surgical castration with subcapsular orchidectomy. Four months after initial diagnosis, he has subsequently developed skeletal metastases and is being treated with radiotherapy to the spine. Case 2 presented with an 8-week history of a rapidly enlarging, maximally 7 cm diameter left deltoid mass. He had a history of T3bN0Mx Gleason score 3 + 4 prostatic adenocarcinoma, had been previously treated with prostate radiotherapy, and was currently being treated with neoadjuvant and adjuvant hormone therapy including leuprorelin acetate. 13 weeks before presentation, he had been administered the first of a 3-year course of 3-monthly injections of subcutaneous leuprorelin acetate, prior to which he had been receiving monthly injection, and his symptoms appeared 2 months after changing from monthly to 3-monthly formulations of the leuprorelin preparation. There was no other past medical history or known history of travel or infection. Serum testosterone at presentation was <0.35 nmol/L (normal range 6.1–27 nmol/L). On examination, there was an approximately 7 cm firm mass over the left biceps muscle, fixed to the underlying structures (Figures 1(a) and 1(b)). Clinically it was of indeterminate nature but was thought to represent a primary soft tissue neoplasm. MRI showed a complex, infiltrative maximally 9 cm lesion in the left deltoid, which extended from subcutaneous to deep tissues to abut the humerus, with underlying normal marrow (Figure 2). This was composed of interlinked foci of abnormal tissue with intense central enhancement, possibly representing necrosis and suggestive of an aggressive inflammatory lesion or sarcoma. The lesion was biopsied.

## 3. Pathology

**Figure 3:**
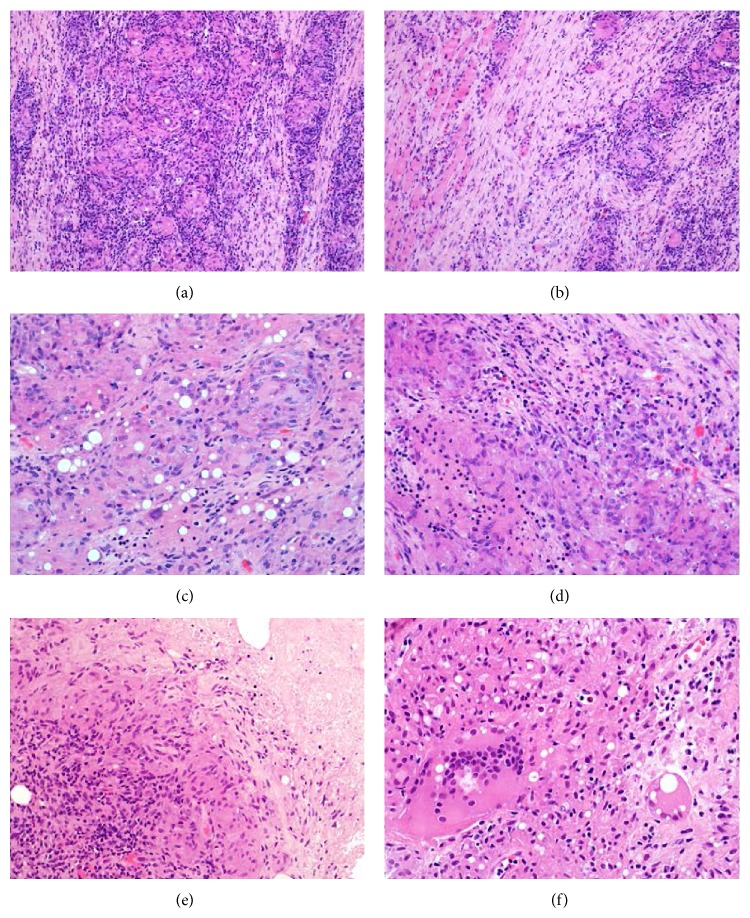
(a) These are relatively ill-defined granulomas comprising sheets of epithelioid histiocytes and giant cells and are surrounded by prominent mixed inflammation rich in small lymphocytes. (b) This example occurred deeply within the biceps muscle. There is atrophy of skeletal muscle fibers, with surrounding fibrosis and chronic inflammation. (c) In areas, there are rounded clear intracytoplasmic vacuoles within the histiocytic population. This is thought to represent foreign body reaction to polylactic acid. Luteinising hormone-releasing hormone (LHRH) has been shown to have lipolytic activity* in vitro*, and the degenerate lipid granules may induce foreign body granulomas. (d) The chronic inflammatory infiltrate comprises largely histiocytes and small lymphocytes, but there are smaller numbers of plasma cells and eosinophils. (e) This example shows areas of necrosis. (f) The giant cells can contain twenty or thirty nuclei. Engulfment of vacuoles corresponding to degenerate elements of leuprorelin acetate is apparent.

Histologically, the core biopsies of both cases showed similar features of fibrous tissue and skeletal muscle with extensive, florid and destructive granulomatous inflammation, composed of relatively ill-defined granulomas containing sheets of epithelioid histiocytes and giant cells, including those of foreign body type (Figures 3(a)–3(f)). The histiocytes had plump, ovoid vesicular nuclei without atypia (Figures 3(a)–3(d)). Case 1 showed no caseation or necrosis, but case 2 showed focal areas of necrosis (Figure 3(e)). No foreign material was identified, but there were prominent rounded clear fatty vacuoles within the histiocytes (Figures 3(c) and 3(f)). There was marked surrounding chronic inflammation, largely composed of small lymphocytes, along with plasma cells and small numbers of eosinophils (Figure 3(d)). The granulomatous inflammation caused prominent skeletal muscle fiber atrophy in case 1 (Figure 3(b)). No neoplasm was identified with desmin, smooth muscle actin (SMA), S100 protein, AE1/AE3, CD117, or CD34. No microorganisms were identified with Ziehl-Neelsen, periodic acid-Schiff or Grocott's stains. In view of the continued clinical suspicion of neoplastic disease, patient 1 had a repeat biopsy, which showed similar findings.

## 4. Discussion

These are rare examples of florid, foreign body granulomas occurring within the subcutis and skeletal muscle of two males in their eighth decades, who were undergoing treatment for prostatic cancer with the luteinizing hormone-releasing hormone analogue leuprorelin acetate. LHRH analogues are widely used in the treatment of diseases that are responsive to the sex hormones, including advanced prostatic carcinoma, breast cancer, endometriosis, and central precocious puberty. When administered continuously, leuprorelin acetate is a potent inhibitor of gonadotropin secretion. After initial gonadotropin stimulation, chronic stimulation with leuprorelin acetate causes downregulation or suppression of these hormones, with subsequent suppression of testicular or ovarian steroidogenesis [1]. LHRH analogues are the mainstay of treatment for advanced prostatic carcinoma, either as primary therapy for metastatic disease or as salvage therapy following surgery or radiation therapy for clinically localized disease [2, 3]. Chemical castration using androgen deprivation therapy with LHRH analogues has been considered equivalent to bilateral orchidectomy in terms of reported testosterone suppression [4]. A sustained-action depot injection system is used to deliver long term controlled chemical castration [5], and to achieve this leuprorelin acetate is coupled to spherical microcapsules made of synthetic biodegradable lactic acid polymers or lactic acid and glycolic acid copolymers [6]. The interior of the polymer matrix contains many fine drug cores containing leuprorelin acetate [6, 7]. This long acting suspension of leuprorelin acetate is injected intramuscularly or subcutaneously and can be given as a monthly injection (3.75 mg) or longer acting 3-monthly preparation (11.25 mg).

There are several known side effects of leuprorelin acetate, including hot flushes, fatigue, nausea and vomiting, loss of libido, and osteoporosis. However, granulomatous nodules occurring at the site of injection are infrequently recognized. While leuprorelin acetate injection site granulomas have been occasionally reported in the urology and dermatology literature [8–13], they are not widely recognized outside these communities. This phenomenon warrants highlighting because of the wide clinical and radiological differential diagnosis, which includes both benign and malignant neoplastic disease and hence the significant potential patient and clinical impact, particularly when lesions are deeply sited (as they have greater propensity to mimic primary or metastatic cancer), which may cause unnecessary patient stress, clinical investigations, or possibly unnecessary surgical intervention [14, 15].

Injection granulomas of leuprorelin acetate are thought to arise from foreign body reaction to polylactic acid [16]. LHRH has been shown to have lipolytic activity* in vitro* [17, 18] and the degenerate lipid granules may induce foreign body granulomas [6]. Granuloma formation may also be dependent on the amount of leuprorelin acetate injected, as most of the reported cases were after injection of the larger 11.25 mg product [5, 6, 19]. Local reactions to depot leuprorelin acetate were first reported in 1992, in the treatment of patients with central precocious puberty [20]. While histology was not described in these cases, “apparent sterile abscess formation” was noted in one patient [20]. Rarely, leuprorelin acetate-induced granulomas have been documented in the arm after subcutaneous injection in the treatment of endometriosis [21], but the overwhelming majority of cases are in men being treated for prostate cancer. While these are still considered to be rare phenomena, many of the reported occurrences are from Japan, where, in a large study of 118 patients who were administered LHRH analogues, there was an incidence of injection site granulomas of 4.2% [19]. Many fewer cases have been reported in Europe and the USA, and authors have hypothesized that the administration of leuprorelin acetate intramuscularly in these areas, but subcutaneously in Japan, might account for the variable geographic incidence of leuprorelin granulomas [19]. The cases we report here occurred within 4 months of each other at our tertiary sarcoma center, but it is difficult to gauge whether this might truly represent an increasing finding in the UK. The reason for the much greater incidence of leuprorelin acetate granulomas occurring in patients with prostate cancer is unclear, although Watanabe et al. postulated that this might be due to the quantitative or qualitative differences in responsiveness of leuprorelin acetate to the LHRH receptor of adipose tissue between men and women [6].

Clinically leuprorelin acetate injection site granulomas tend to manifest as firm, often multiple erythematous nodules measuring approximately 2–6 cm [1, 21], often with a suppurative appearance [22], in the subcutis or sometimes skeletal muscle. The duration from time of first injection to onset of clinical findings varies from 35 to 350 days (mean 150 days), and it has been reported that most granulomas occurred after the first or second administration of the 11.25 mg quantity [19]. Development of granulomas is also noted to occur after the depot type was changed from a 1-monthly to 3-monthly formulation [1]. The clinical differential diagnosis is wide, ranging from inflammatory or reparative lesions such as traumatic panniculitis or hematoma or infective nodules from bacterial, mycobacterial, or fungal infection [21] to benign and malignant neoplasms. Because of their rapid enlargement, they can be mistaken for metastatic deposits [9, 14] or soft tissue sarcoma, such as epithelioid sarcoma, which can present similarly as numerous erythematous nodules at an extremity site. Alternatively, leuprorelin acetate granulomas can be mistaken for primary benign soft tissue neoplasms associated with rapid growth, such as nodular fasciitis or myositis ossificans.

Histologically, there are prominent granulomas usually centered within the subcutis but sometimes seen within the dermis or skeletal muscle and composed of sheets and nodules of epithelioid histiocytes with foreign body giant cells [6]. The histiocytes typically contain numerous translucent intracytoplasmic vacuoles of varying sizes, as well as variable degeneration of the adipose tissue. Sometimes the granulomas have a palisading appearance with eosinophils, and occasionally there may be central necrosis and abscess formation [6]. It is important not to miss a necrotic neoplasm, however, so immunohistochemistry with a basic panel of markers (AE1/AE3, desmin, SMA, CD34, S100 protein, and CD45/CD20/CD3) is likely to be contributory to excluding this. Ultrastructurally, within the cells of the granulomas, there are electron-lucent spherical bodies conforming to microcapsules of leuprorelin acetate products, as well as needle-shaped crystalloid structures in lipid droplets (degenerated lipid droplets) within both the granuloma cells and adipose tissue [6].

The phenomenon of leuprorelin acetate-induced injection site granulomas is still relatively little known outside the fields of urology and dermatology, and, with the increasing subspecialization of medicine, lesional biopsy may be performed at a tertiary sarcoma or orthopedic center, rather than the patient's local hospital, without access to or knowledge of the patient's medical history. This highlights the importance of clinical correlation and diagnostic recognition, in order to avoid misdiagnosis of these generally self-limiting lesions as malignant neoplasms.
